# Osteopontin–integrin engagement induces HIF-1α–TCF12-mediated endothelial-mesenchymal transition to exacerbate colorectal cancer

**DOI:** 10.18632/oncotarget.23578

**Published:** 2017-12-22

**Authors:** Chi-Shuan Fan, Wei-Shone Chen, Li-Li Chen, Chia-Chi Chen, Yu-Ting Hsu, Kee Voon Chua, Horng-Dar Wang, Tze-Sing Huang

**Affiliations:** ^1^ Institute of Biotechnology, National Tsing-Hua University, Hsinchu, Taiwan; ^2^ National Institute of Cancer Research, National Health Research Institutes, Miaoli, Taiwan; ^3^ Division of Colorectal Surgery, Taipei Veterans General Hospital, and Department of Medicine, National Yang-Ming University, Taipei, Taiwan; ^4^ Department of Biochemistry, School of Medicine, Kaohsiung Medical University, Kaohsiung, Taiwan

**Keywords:** EndoMT, HIF-1α, TCF12, eHSP90α, cancer cell stemness

## Abstract

Osteopontin (OPN) is a multi-functional phospho-glycoprotein that can stimulate angiogenesis through acting on endothelial cells. As angiogenic sprouting involves endothelial-to-mesenchymal transition (EndoMT), we are intrigued to know whether OPN exerts an effect on EndoMT. Clinically, we indeed detected EndoMT-derived cells next to OPN-expressing cells in colorectal cancer tissues. Furthermore, we treated OPN to primary cultures of endothelial cells to investigate the EndoMT-inducing activity and the underlying mechanisms. Integrin α_V_β_3_ rather than CD44 is involved in OPN-induced EndoMT. OPN-integrin α_V_β_3_ engagement induces HIF-1α expression through a PI3K/Akt/TSC2-mediated and mTORC1-dependent protein synthesis pathway, which in turn trans-activates *TCF12* gene expression. TCF12 further interacts with EZH2 and histone deacetylases to transcriptionally repress *VE-cadherin* gene and thus facilitates EndoMT. Like cancer-associated fibroblasts, EndoMT-derived cells promote tumor growth and metastasis by secreting certain proteins. Secreted HSP90α is a candidate suggested by microwestern array assay, and is herein verified to induce stemness properties in colorectal cancer cells. As OPN is overexpressed in human cancers, OPN-induced EndoMT and EndoMT-derived cells can be potentially taken as cancer therapeutic targets.

## INTRODUCTION

Cell plasticity is essential for many mature functional cells to retain the potential to de-differentiate back to stem/progenitor cells or to trans-differentiate into other distant cell lineages [1]. As a result, it provides various cell types needed for tissue microenvironmental reprogramming upon physiological or pathological demands. Endothelial-to-mesenchymal transition (EndoMT) is a remarkable example of cell plasticity widely observed in embryonic heart formation [2–4] and diseases like cardiac fibrosis [5], atherosclerosis [6], pulmonary hypertension [7], and cancer development [8]. With regard to cancer, EndoMT can be a source of 30–40% of cancer-associated fibroblasts (CAFs) [8] which facilitate malignant progression by secreting growth factors and extracellular matrix molecules [9]. EndoMT can also play an important role in tumor angiogenesis. It is thought to participate in vascular sprouting, allowing the tip cells to migrate/invade into adjacent tissue [10]. Additionally, vessel-supporting cells such as pericytes and smooth muscle cells may arise from endothelial cells (ECs) through EndoMT [11].

EndoMT was first observed in cardiac organogeny [2–4] and many understandings of EndoMT are based on the studies of heart development. However, there is a substantial phenotypic variability in ECs throughout the whole vascular architectures [12]; especially in disease states, the ECs can particularly be changed within different tissue microenvironments to meet different pathologic needs. For example, TGF-β induces a Smad3-mediated EndoMT during cardiac fibrosis [5], but a Smad3-independent signaling pathway in pulmonary ECs [13]. Slug expression is involved in cardiac EndoMT, but in pulmonary ECs, TGF-β treatment up-regulated Snail instead of Slug [13]. Inhibitory phosphorylation of GSK-3β by PKC-δ and c-Abl, which avoids the phosphorylation and degradation of Snail, contributes to a non-Smad pathway induced by TGF-β in pulmonary but not in cardiac ECs [14]. Overall, intimate knowledge of EndoMT in different diseases remains elusive, especially in terms of cancers. In contrast to the extensive studies on epithelial-to-mesenchymal transition (EMT), there is still a paucity of information about the EndoMT process in cancers, and there are only very few studies have explored the molecular mechanisms involved in cancer-related EndoMT.

Hypoxia-inducible factor-1α (HIF-1α), first identified as a regulator of erythropoietin, is required for embryonic cardiac and vascular development and erythropoiesis [15–17]. It is an important transcription factor responsive to hypoxic stress and induces target gene expressions to mediate cellular adaptive responses. Under normoxia, HIF-1α is hydroxylated by prolyl hydroxylases and is subsequently ubiquitylated with the aids of von Hippel-Lindau protein (VHL) and cullin2 E3 ligase, which eventually leads to degradation by 26S proteasomes [18]. Hypoxia increases mitochondrial reactive oxygen species that inactivates prolyl hydroxylases through oxidation of ferrous ions, and thus causes HIF-1α accumulation [19]. Besides the stability regulation, HIF-1α protein synthesis can be activated in cancer cells through the phosphatidylinositol-3-kinase (PI3K)–Akt signaling axis primarily by the action of mammalian target of rapamycin complex 1 (mTORC1) [20, 21]. Tuberous sclerosis complex 2 (TSC2) is an inhibitory protein of mTORC1 by forming a complex with TSC1. However, Akt can phosphorylate TSC2 and thereby disrupts TSC1–TSC2 complex formation [21]. Once HIF-1α is increased, it can promote cancer cell EMT by inducing expressions of downstream genes like Twist-1 and ZEB1 [22, 23]. Additionally, it also induces lysyl oxidases LOXL2/3 to stabilize Snail [24]. Twist-1, ZEB1/2, and Snail are all transcriptional repressors of E-cadherin and thus promote cancer cell EMT. Nonetheless, it remains uncertain whether HIF-1α and its downstream targets are also involved in cancer-related EndoMT.

TCF12 is a class-1 member of the helix-loop-helix protein family and functions as a transcriptional repressor of E-cadherin like Twist-1, ZEB1/2, and Snail [25]. TCF12 not only forms a homodimer and directly binds to the E-box sites of target gene promoters, but also forms a heterodimer with other class member like Twist-1. TCF12 was observed to be co-expressed and co-immunoprecipitated with Bmi1 and EZH2 in human colorectal cancer (CRC) cells [25]. It was also reportedly associated with EZH2, JARID2, and histone H3 trimethyllysine-27 (H3K27me3) in mouse embryonic stem cells and involved in developmental control [26]. These studies suggest that TCF12 acts as a transcriptional repressor together with polycomb group-repressive complex PRC1 and PRC2. Overexpression of TCF12 in CRC cells is associated with increased levels of cellular EMT, migration, invasion, and metastasis [25]. Clinically, elevated TCF12 expression was detected in the tumor tissues of CRC patients with a significantly higher rate of metastasis. Additionally, higher serum HSP90α levels were measured from CRC patients with an elevation of TCF12 expression in their tumor tissues [27]. Extracellular HSP90α (eHSP90α) can be a naturally occurring inducer of TCF12 overexpression in tumor. This proposition was evidenced by an *in vitro* study showing that eHSP90α induced TCF12 expression through a NF-κB-mediated pathway [27]. Besides EMT, we wonder if TCF12 is involved in EndoMT.

Osteopontin (OPN) is a phosphorylated glycoprotein originally identified as a bone matrix protein and is subsequently thought as a cytokine participated in many physiological and pathological processes including bone turnover, immune responses, wound healing, ischemia as well as tumor development and progression [28]. OPN can be expressed by many different cell types, including macrophages, ECs, and neoplastic epithelial cells. OPN is overexpressed in human cancers, and elevated serum/plasma OPN levels are substantially correlated with high metastatic occurrence and poor prognosis [29]. Through binding to cell receptors integrin α_V_β_3_and CD44, OPN not only promotes tumor cell survival, proliferation, migration, and invasion, but also acts on ECs to stimulate tumor angiogenesis and metastasis [29, 30]. As angiogenic sprouting involves EndoMT, the effect of OPN on EndoMT and the underlying mechanisms need to be fully investigated.

In this study, OPN shows EndoMT-inducing activity. By interacting with integrin α_V_β_3_ but not CD44, OPN induces a PI3K/Akt/TSC2-mediated and mTORC1-dependent protein synthesis of HIF-1α which in turn trans-activates *TCF12* gene expression. Furthermore, TCF12 interacts with EZH2 and histone deacetylases (HDACs) to act as a transcriptional repressor of *VE-cadherin* gene and thus facilitates EndoMT. Like CAFs, EndoMT-derived cells secrete certain proteins to promote tumor growth and metastasis of CRC cell xenografts. eHSP90α is an example protein exhibiting the ability to induce CRC cell stemness.

## RESULTS

### OPN induces EndoMT

**Figure 1:**
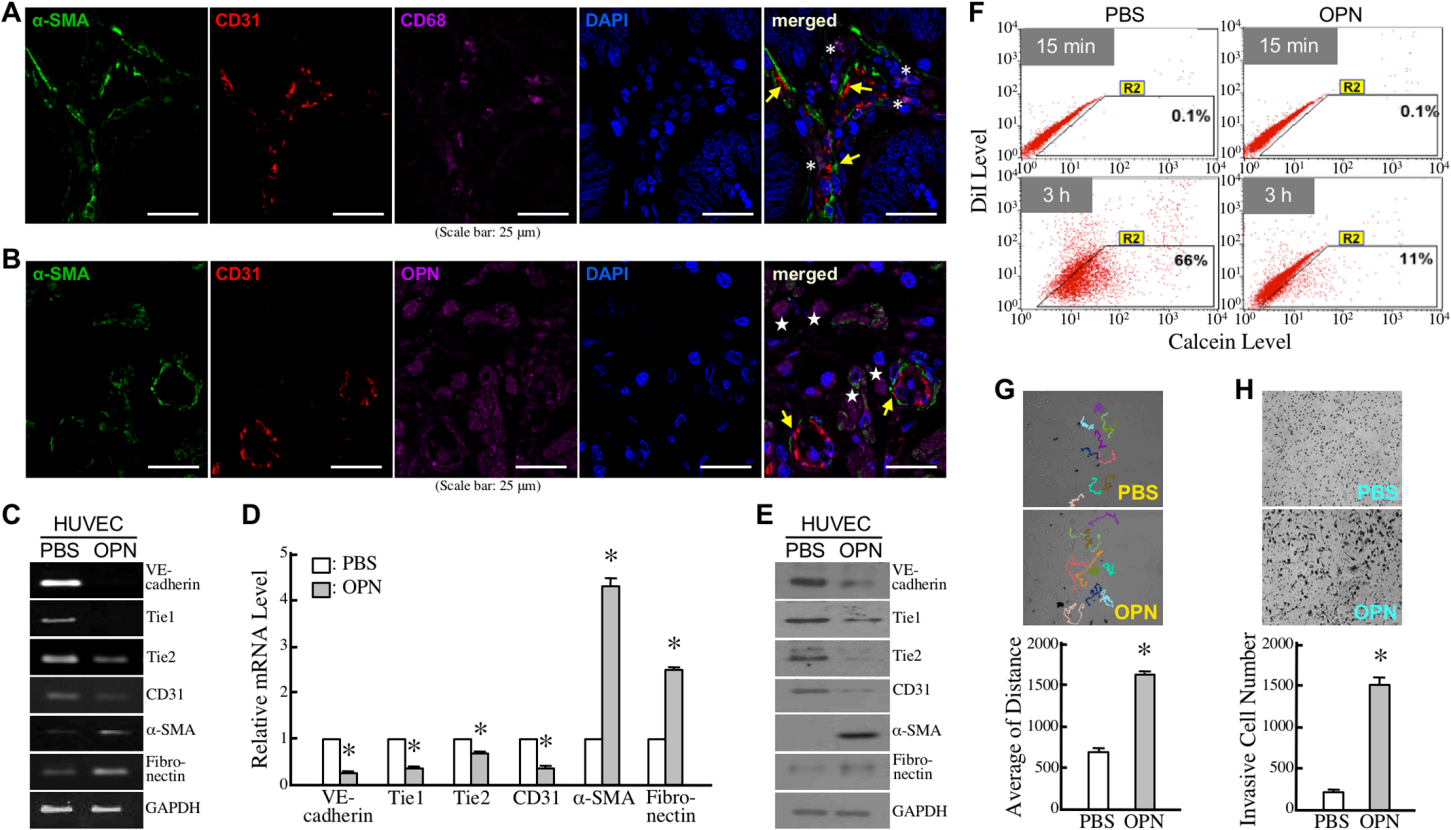
OPN induces EndoMT **(A)** Immunohistofluorescent staining of CD68, α-SMA, and CD31 showing EndoMT-derived cells and neighboring macrophages in CRC tissues. Examples of EndoMT-derived cells and macrophages were indicated by arrows and ^*^, respectively. **(B)** Immunohistofluorescent staining of OPN, α-SMA, and CD31 showing EndoMT-derived cells and OPN-expressing cells in CRC tissues. Examples of EndoMT-derived cells and OPN-expressing cells were indicated by arrows and stars, respectively. **(C)** mRNA levels of VE-cadherin, Tie1, Tie2, CD31, α-SMA, and fibronectin in HUVECs pre-incubated 16 h with 2% FBS-containing medium and then treated 15 h with PBS or 0.3 μg/ml of OPN. **(D)** Quantification of VE-cadherin, Tie1, Tie2, CD31, α-SMA, and fibronectin mRNA levels in HUVECs treated as described in (C). The mean ± SD values of 3 independent experiments are shown. ^*^, *P* < 0.05 when compared with the data of PBS-treated cells. **(E)** VE-cadherin, Tie1, Tie2, CD31, α-SMA, and fibronectin levels in HUVECs pre-incubated 16 h with 2% FBS-containing medium and then treated 24 h with PBS or 0.3 μg/ml of OPN. **(F)** Gap-junction activities in HUVECs treated with PBS or OPN as described in (E). Representative dot plots from 3 independent Calcein-transfer assays are shown. The cells in the R2 regions were categorized as Calcein-accepting cells. The % of Calcein-accepting cells quantified by CellQuest software represent cellular gap-junction activities of the tested cells. **(G)** Cell migration activities in HUVECs treated with PBS or OPN as described in Materials and Methods. Cell migration was monitored for 15 h using time-lapse photography, and the movement tracks of 10 randomly selected PBS or OPN-treated HUVECs were analyzed by Image-Pro Plus software. Quantification of the accumulated migration distances is shown in the bottom panel. The data are the mean ± SD values of 3 independent experiments. ^*^, *P* < 0.05 when compared with PBS-treated cells. **(H)** Invasiveness of PBS or OPN-treated HUVECs determined by Transwell invasion assay. The representative images shown were invasive cells on the filters of Transwell inserts. The mean ± SD values of 3 independent experiments are shown in the bottom panel. ^*^, *P* < 0.05 when compared with PBS-treated cells.

In CRC tissues, we detected the EndoMT-derived cells exhibiting α-SMA^+^/CD31^+^ in neighboring macrophages (Figure 1A). Because macrophages are a major cell source of OPN, we also detected α-SMA^+^/CD31^+^ EndoMT-derived cells nearby OPN-expressing cells (Figure 1B). Therefore, we wondered if OPN exerted any effect on EndoMT. After serum starvation with 2% FBS-containing medium for 16 h, HUVECs were treated with PBS or 0.3 μg/ml of OPN for another 15 h. The mRNA levels of VE-cadherin, Tie1, Tie2, and CD31 were reduced in OPN-treated HUVECs, whereas those of cellular α-SMA and fibronectin were up-regulated simultaneously (Figure 1C, 1D). Additionally, both reduction of VE-cadherin, Tie1, Tie2, and CD31 and induction of α-SMA and fibronectin were detected at protein levels in HUVECs treated with OPN for 24 h (Figure 1E). By down-regulating VE-cadherin expression, OPN showed a repressive effect on cell-cell junctions of HUVECs, which was demonstrated by reduction of cellular gap-junction activity measured by Calcein transfer assay (Figure 1F). In contrast, OPN exhibited enhancing effects on cell migration and invasion activities of HUVECs (Figure 1G, 1H). The OPN effects were also observed in EC-RF24 cells, an immortalized EC line (Supplementary Figure 1). Taken together, these data suggest that OPN induces EndoMT.

### TCF12 is involved in OPN-induced EndoMT

**Figure 2:**
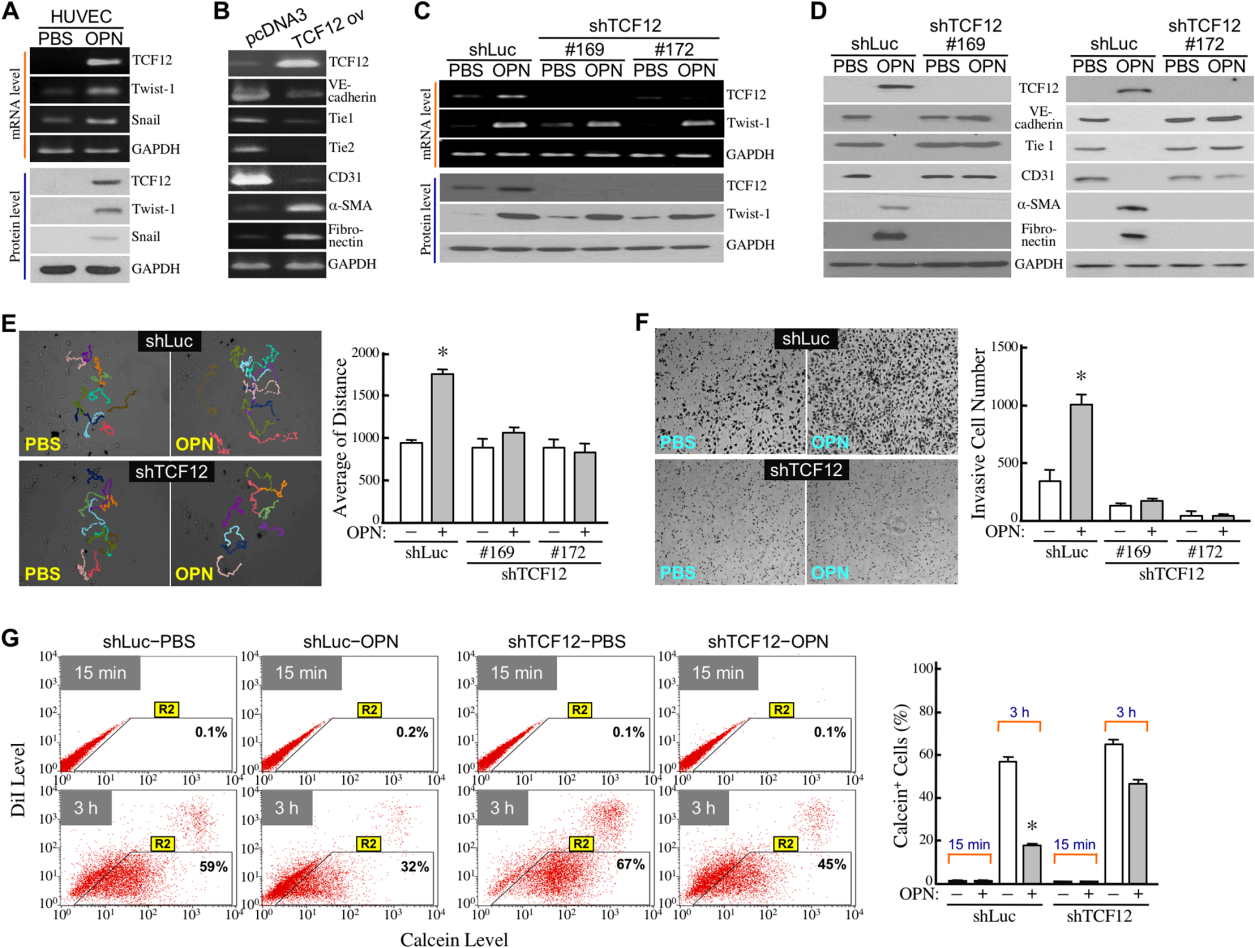
TCF12 is involved in OPN-induced EndoMT **(A)** mRNA and protein levels of TCF12, Twist-1, and Snail in HUVECs treated with PBS or 0.3 μg/ml of OPN as described previously. **(B)** mRNA levels of TCF12, VE-cadherin, Tie1, Tie2, CD31, α-SMA, and fibronectinin EC-RF24 cells transfected with empty vector control (pcDNA3) or TCF12-expressing recombinant plasmid (TCF12 ov). **(C)** mRNA and protein levels of TCF12 and Twist-1 in PBS or OPN-treated HUVECs expressing control shRNA (shLuc) or TCF12 shRNA #169 and #172, respectively. **(D)** mRNA and protein levels of TCF12, VE-cadherin, Tie1, CD31, α-SMA, and fibronectin levels in control and TCF12-knockdown HUVECs treated with PBS or OPN. **(E)** Cell migration activities of control and TCF12-knockdown HUVECs treated with PBS or OPN. The movement tracks of 10 randomly selected cells in each group were analyzed by Image-Pro Plus software. The accumulated migration distances were quantified and the mean ± SD values of 3 independent experiments are shown in the right panel. ^*^, *P* < 0.05 when compared with PBS-treated cells. **(F)** Invasiveness of control and TCF12-knockdown HUVECs treated with PBS or OPN. The mean ± SD values of 3 independent Transwell invasion assays are shown in the right panel. ^*^, *P* < 0.05 when compared with PBS-treated cells. **(G)** Gap-junction activities in control and TCF12-knockdown HUVECs treated with PBS or OPN. The mean ± SD values of 3 independent Calcein-transfer assays are shown. The % of Calcein-accepting cells (designated as “Calcein^+^ cells”) represents the gap-junction activity of tested cells. ^*^, *P* < 0.05 when compared with PBS-treated cells.

To disclose the molecular mechanisms responsible for OPN-induced EndoMT, we first investigated the effects of OPN on the expression of EMT-inducing transcriptional factors TCF12, Twist1, and Snail in HUVECs. We observed that TCF12, Twist1, and Snail were all increased at both mRNA and protein levels in OPN-treated HUVECs (Figure 2A). Unlike Snail, roles of TCF12 and Twist1 in EndoMT have not yet been reported. We have previously shown that TCF12 is a transcriptional factor that can directly and specifically bind to the E-box sites of target gene promoters. Twist1 is a class-2 member of the helix-loop-helix protein family and its transcription-regulatory activity requires the interaction with a class-1 protein. Therefore, we focused on the study of TCF12 involvement in OPN-induced EndoMT. When TCF12 was ectopically overexpressed in EC-RF24 cells, cellular VE-cadherin, Tie1, Tie2, and CD31 levels were reduced, whereas α-SMA and fibronectin expressions were up-regulated (Figure 2B). On the other hand, ectopic expression of TCF12 shRNAs could effectively suppress basal and OPN-induced TCF12 mRNA and protein levels without significantly affecting Twist1 expression in HUVECs (Figure 2C). In these TCF12-knockdown cells, OPN could no longer induce down-regulation of VE-cadherin, Tie1, and CD31 levels and up-regulation of α-SMA and fibronectin levels (Figure 2D). Both cell migration and invasion induced by OPN were also drastically abolished (Figure 2E, 2F). Additionally, the repressive effect of OPN on cellular gap-junction activity was diminished when TCF12 expression was knocked down (Figure 2G). These data suggest that TCF12 is required for OPN-induced EndoMT.

### TCF12 functions as a transcriptional repressor of *VE-cadherin* gene

**Figure 3:**
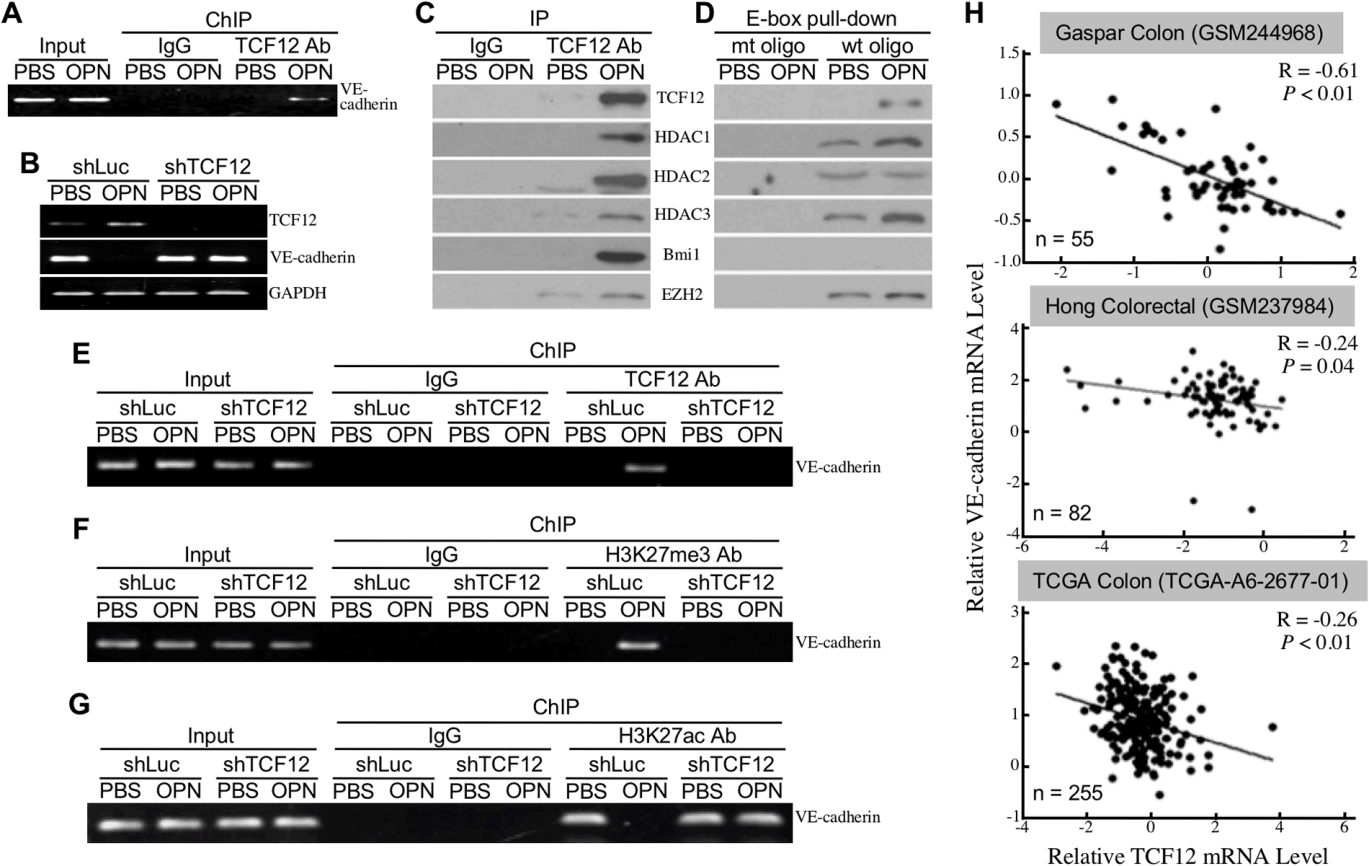
TCF12 functions as a transcriptional repressor of VE-cadherin **(A)** ChIP assay showing thephysical association of TCF12 with the E-box site (_-430_CAGCTG_-425_) of *VE-cadherin* gene promoter induced by OPN in HUVECs. **(B)** mRNA levels of TCF12 and VE-cadherin in control and TCF12-knockdown HUVECs treated with PBS or OPN. **(C)** TCF12, HDAC1, HDAC2, HDAC3, Bmi1, and EZH2 levels in the control IgG and anti-TCF12 immunoprecipitates from HUVECs treated with PBS or 0.3 μg/ml of OPN as described previously for 24 h. **(D)** TCF12, HDAC1, HDAC2, HDAC3, Bmi1, and EZH2 levels in the E-box-pulled down proteins from HUVECs treated the same as in (C). The biotin-conjugated oligonucleotides containing the wild-type or mutant E-box site of *VE-cadherin* gene (designated as “wt oligo” and “mt oligo”, respectively) were synthesized and incubated with the nuclear extracts of PBS or OPN-treated HUVECs. Streptavidin-conjugated Sepharose beads were added into the samples to pull down E-box-associated proteins through centrifugation. The precipitates were assayed by immunoblotting with antibodies against TCF12, HDAC1, HDAC2, HDAC3, Bmi1, and EZH2. **(E-G)** ChIP assay showing the physical association of TCF12 with *VE-cadherin* gene promoter (E), the physical association of H3K27me3 with *VE-cadherin* gene promoter (F), and a dissociation of H3K27ac from *VE-cadherin* gene promoter (G) induced by OPN in control but not in TCF12-knockdown HUVECs. **(H)** A trend of reverse correlation between the levels of VE-cadherin mRNA and TCF12 mRNA was noted from three publicized datasets GSM244968, GSM237984, and TCGA-A6-2677-01.

TCF12 functions as a transcriptional repressor of *E-cadherin* gene through binding to the E-box sites of *E-cadherin* gene promoter region. Because putative E-box sites can also be found on the promoter region of *VE-cadherin* gene, TCF12 was proposed to be a transcriptional repressor of *VE-cadherin* gene. Using chromatin immunoprecipitation (ChIP) assay, we confirmed that OPN induced an association of TCF12 with the E-box-containing promoter region of *VE-cadherin* gene (Figure 3A). Moreover, OPN-induced down-regulation of VE-cadherin mRNA expression was drastically suppressed when TCF12 was knocked down in HUVECs (Figure 3B). To study if TCF12 formed complexes with transcriptional repression machineries PRC1 and PRC2, we performed immunoblot analyses to detect the presence of Bmi1, EZH2, and HDACs in anti-TCF12 immunoprecipitates. As OPN induced TCF12 level, the TCF12-associated Bmi1, EZH2, HDAC1, HDAC2, and HDAC3 levels were also increased in OPN-treated HUVECs (Figure 3C). Based on Figure 3A, we synthesized a pair of biotin-conjugated oligonucleotides containing the E-box site of *VE-cadherin* gene promoter and incubated it with the nuclear extracts of PBS or OPN-treated HUVECs. After the addition of Streptavidin-conjugated Sepharose beads, the E-box-associated proteins were pulled down and further analyzed for the existence of Bmi1, EZH2, HDAC1, HDAC2, and HDAC3. As shown in Figure 3D, OPN induced levels of TCF12, HDAC1, HDAC3, and EZH2 that physically associated with the E-box site of *VE-cadherin* gene. Although Bmi1 was co-immunoprecipitated with TCF12 (Figure 3C), it was not associated with the E-box site of *VE-cadherin* gene either directly or indirectly via TCF12 (Figure 3D). Given that EZH2 is a histone-lysine N-methyltransferase, we investigated whether the E-box-containing promoter region of *VE-cadherin* gene was wrapped by hypermethylated histones. We performed ChIP assays using antibodies against H3K27me3, histone H3 acetyllysine-27 (H3K27ac), and TCF12. The results revealed that OPN significantly induced associations of the E-box-containing region with H3K27me3 as well as TCF12 (Figure 3E, 3F). In contrast, OPN induced a TCF12-dependent dissociation of H3K27ac from the region (Figure 3G). Taken together, these data suggested that TCF12 functioned as a transcriptional repressor of *VE-cadherin* gene with an aid of EZH2-associated histone H3 hypermethylation. Clinically, a trend of reverse correlation between TCF12 and VE-cadherin mRNA levels was also observed from CRC tissues after analyses of three publicized datasets TCGA-A6-2677-01, GSM244968, and GSM237984 (Figure 3H).

### OPN induces TCF12 expression through integrin α_V_β_3_-PI3K-Akt signaling axis

**Figure 4:**
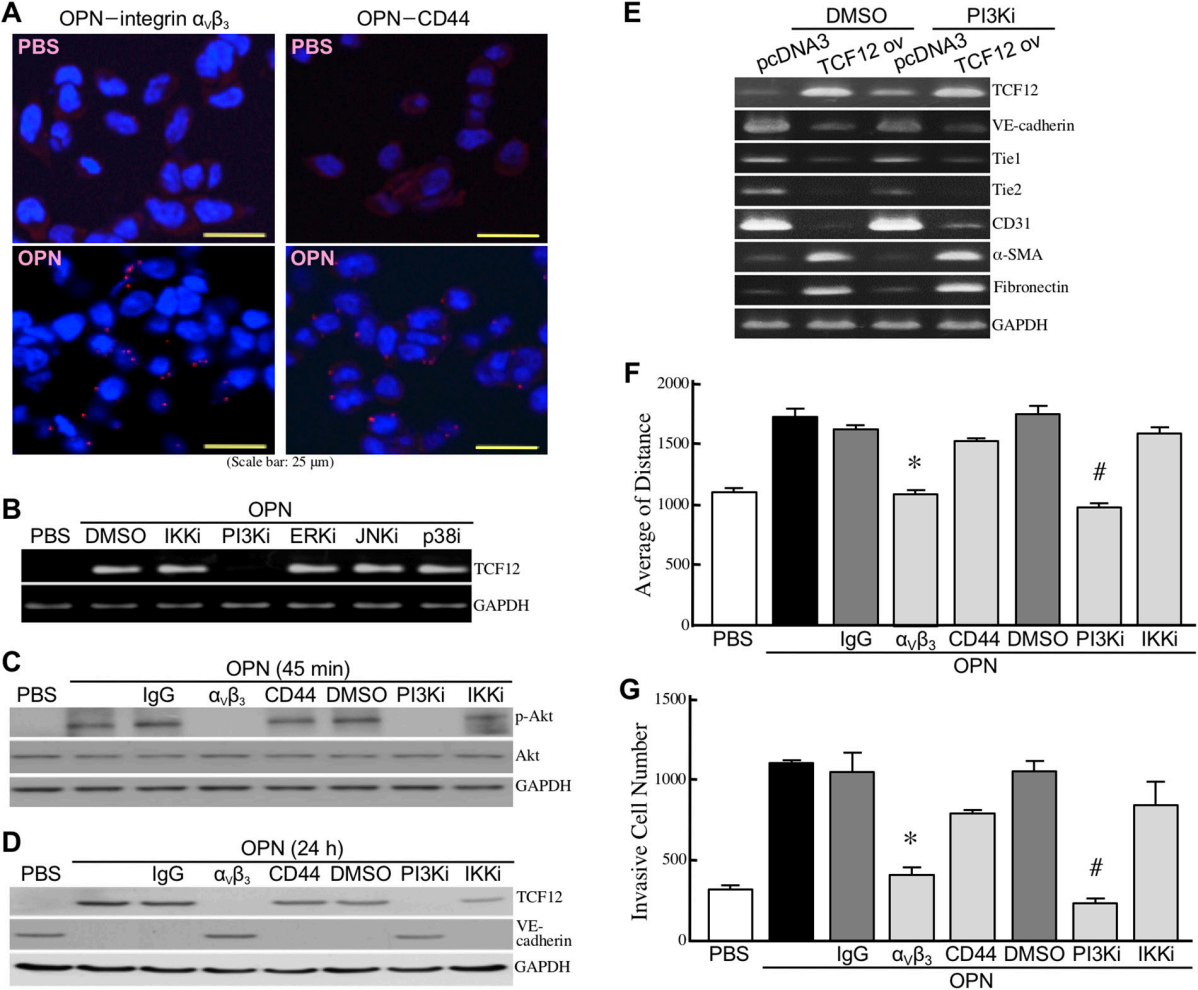
OPN induces TCF12 expression via the integrin α_V_β_3_-PI3K-Akt pathway **(A)** PLA showing physical associations of OPN with integrin α_V_β_3_ and CD44 in HUVECs. HUVECs were treated with PBS or OPN as described previously for 45 min, and double-stained with anti-OPN and anti-integrin α_V_β_3_ antibodies or with anti-OPN and anti-CD44 antibodies. Red fluorescent dots represent direct contacts of OPN with integrin α_V_β_3_ or CD44. **(B)** mRNA levels of TCF12 in HUVECs treated with PBS or OPN as described previously for 15 h in the absence or presence of the inhibitor of IKKα/β, PI3K, ERK, JNK, or p38. **(C)** Phospho-Akt and total Akt levels in HUVECs pre-incubated with 2% FBS-containing medium for 16 h and treated 45 min with PBS, 0.3 μg/ml of OPN, or 0.3 μg/ml of OPN with or without the antibody against integrin α_V_β_3_ or CD44 or the inhibitor against PI3K or IKKα/β. **(D)** TCF12 and VE-cadherin levels in HUVECs treated as described in (C) for 24 h. **(E)** mRNA levels of TCF12, VE-cadherin, Tie1, Tie2, CD31, α-SMA, and fibronectinin the control or TCF12-overexpressing EC-RF24 cells treated 24 h with DMSO or 50 μM of PI3K inhibitor LY294002. **(F)** Cell migration activities of HUVECs treated with PBS, 0.3 μg/ml of OPN, or 0.3 μg/ml of OPN with or without the antibody against integrin α_V_β_3_ or CD44 or the inhibitor of PI3K or IKKα/β. The data are expressed as mean ± SD values of 3 independent experiments. ^*^, *P* < 0.05 when compared with the treatment with OPN plus IgG. ^#^, *P* < 0.05 when compared with the treatment with OPN plus DMSO. **(G)** Invasiveness of HUVECs treated with PBS, 0.3 μg/ml of OPN, or 0.3 μg/ml of OPN with or without the antibody against integrin α_V_β_3_ or CD44 or the inhibitor of PI3K or IKKα/β. The mean ± SD values of 3 independent experiments are shown. ^*^, *P* < 0.05 when compared with the treatment with OPN plus IgG. ^#^, *P* < 0.05 when compared with the treatment with OPN plus DMSO.

To investigate whether integrin α_V_β_3_ and CD44 served as cellular receptors for OPN in HUVECs, we performed proximity ligation assay (PLA) and found that significant amounts of red fluorescence dots representing OPN-integrin α_V_β_3_ or OPN-CD44 associations were induced on OPN-treated HUVECs (Figure 4A). Next, we studied the underlying signaling pathway(s) for OPN-induced TCF12 expression. We found that OPN-induced TCF12 mRNA expression was drastically abolished by PI3K inhibitor but not by IKK, ERK, JNK, and p38 inhibitors (Figure 4B). OPN-induced phosphorylated Akt and TCF12 levels were also significantly suppressed by PI3K inhibitor and the antibody against integrin α_V_β_3_ but not CD44 (Figure 4C, 4D), suggesting that OPN induced TCF12 expression via an integrin α_V_β_3_- and PI3K/Akt-mediated signaling pathway. Moreover, induction of EndoMT markers by ectopic TCF12 overexpression was not affected by PI3K inhibitor (Figure 4E), confirming that TCF12 is a major downstream effector of OPN-induced PI3K/Akt pathway that is involved in EndoMT. Inhibition of the integrin α_V_β_3_-PI3K-Akt signaling pathway efficiently suppressed the activities of cell migration and invasion in OPN-treated HUVECs (Figure 4F, 4G).

### HIF-1α is involved in OPN-induced TCF12 expression

**Figure 5:**
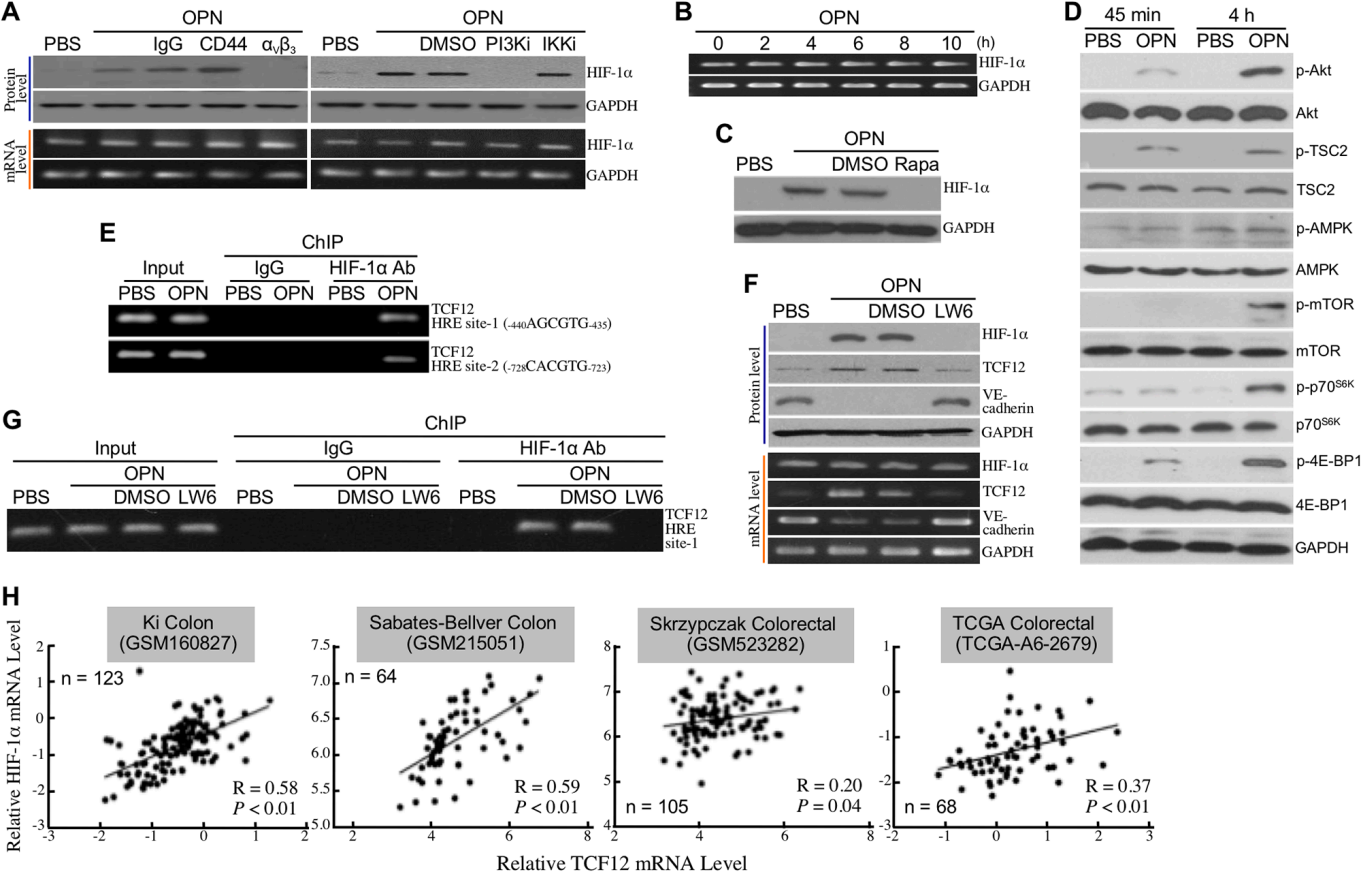
HIF-1α is involved in OPN-induced TCF12 expression **(A)** mRNA and protein levels of HIF-1α in HUVECs pre-incubated 16 h with 2% FBS-containing medium and treated 12 h with PBS, 0.3 μg/ml of OPN, or 0.3 μg/ml of OPN with or without the antibody against integrin α_V_β_3_ or CD44 or the inhibitor of PI3K or IKKα/β. **(B)** mRNA levels of HIF-1α in HUVECs treated with OPN as described previously for 2, 4, 6, 8, or 10 h. **(C)** HIF-1α levels in HUVECs treated with OPN as described previously for 12 h in the absence or presence of rapamycin. **(D)** Phospho-Akt, Akt, phospho-TSC2, TSC2, phospho-AMPK, AMPK, phospho-mTOR, mTOR, phospho-p70^S6K^, p70^S6K^, phospho-4E-BP1, and 4E-BP1 levels in HUVECs treated with PBS or OPN as described previously for 45 min or 4 h. **(E)** ChIP assay showing the physical association of HIF-1α with *TCF12* gene promoter in HUVECs treated with OPN as described previously for 12 h. Two HRE sites, -440 to -435 and -728 to -723, in *TCF12* gene promoter were shown to be associated with HIF-1α. **(F)** mRNA and protein levels of HIF-1α, TCF12, and VE-cadherin in HUVECs treated with OPN as described previously for 12 h in the absence or presence of LW6. **(G)** ChIP assay showing disruption of the physical association of HIF-1α with *TCF12* gene promoter (HRE site-1) in HUVECs treated with OPN in the presence of LW6. **(H)** A trend of positive correlation between the levels of HIF-1α mRNA and TCF12 mRNA was noted from four publicized datasets GSM160827, GSM215051, GSM523282, and TCGA-A6-2679.

As mentioned above, HIF-1α is required for cardiac and vascular development and is a transcriptional factor responsive to PI3K–Akt activity in cancer cells. It was therefore postulated to be involved in OPN-induced EndoMT. We found that HIF-1α was induced at protein level but not at mRNA level via an integrin α_V_β_3_- and PI3K/Akt-mediated signaling pathway in HUVECs after a 12-h stimulation with OPN (Figure 5A). The mRNA level of HIF-1α was not significantly altered by OPN treatment within 10 h (Figure 5B). Induction of HIF-1α protein by OPN was sensitive to rapamycin (Figure 5C), suggesting that this induction was through an mTORC1-dependent *de novo* protein synthesis. Indeed, OPN induced both activating phosphorylation of mTOR and p70^S6K^ and inactivating phosphorylation of 4E-BP1, which was consistent with the result that OPN induced activating phosphorylation of Akt and inactivating phosphorylation of TSC2 (Figure 5D). Because the DNA binding sites of HIF-1α (HREs) can be found on *TCF12* gene promoter, we performed ChIP assay to investigate if OPN induced physical associations of HIF-1α with the putative HREs. Two putative HREs _–440_AGCGTG_–435_ and _–728_CACGTG_–723_ were found to be bound by HIF-1α in OPN- but not in PBS-treated HUVECs (Figure 5E). LW6 is a potent HIF-1α suppressor through enhancing HIF-1α degradation. When HUVECs were treated with OPN in the presence of LW6, cellular TCF12 induction by OPN was abolished and VE-cadherin down-regulation was recovered (Figure 5F). In the HUVECs treated with OPN together with LW6, OPN-induced HIF-1α level was suppressed by LW6 and therefore the HRE-containing promoter region could not be detected in ChIP assay (Figure 5G). Taken together, these data suggest that HIF-1α is a transcription factor responsible for OPN-induced integrin α_V_β_3_-PI3K-Akt-mediated TCF12 mRNA expression. Using *in silico* analyses of publicized datasets, a trend of positive correlation was also observed between HIF-1α and TCF12 mRNA levels in the tissue specimens of CRC patients (Figure 5H). Accordingly, we also found that LW6 could suppress OPN-inhibited cellular gap-junction activity as well as OPN-induced cell migration and invasion in HUVECs (Supplementary Figure 2).

### EndoMT enhances CRC malignancy

**Figure 6:**
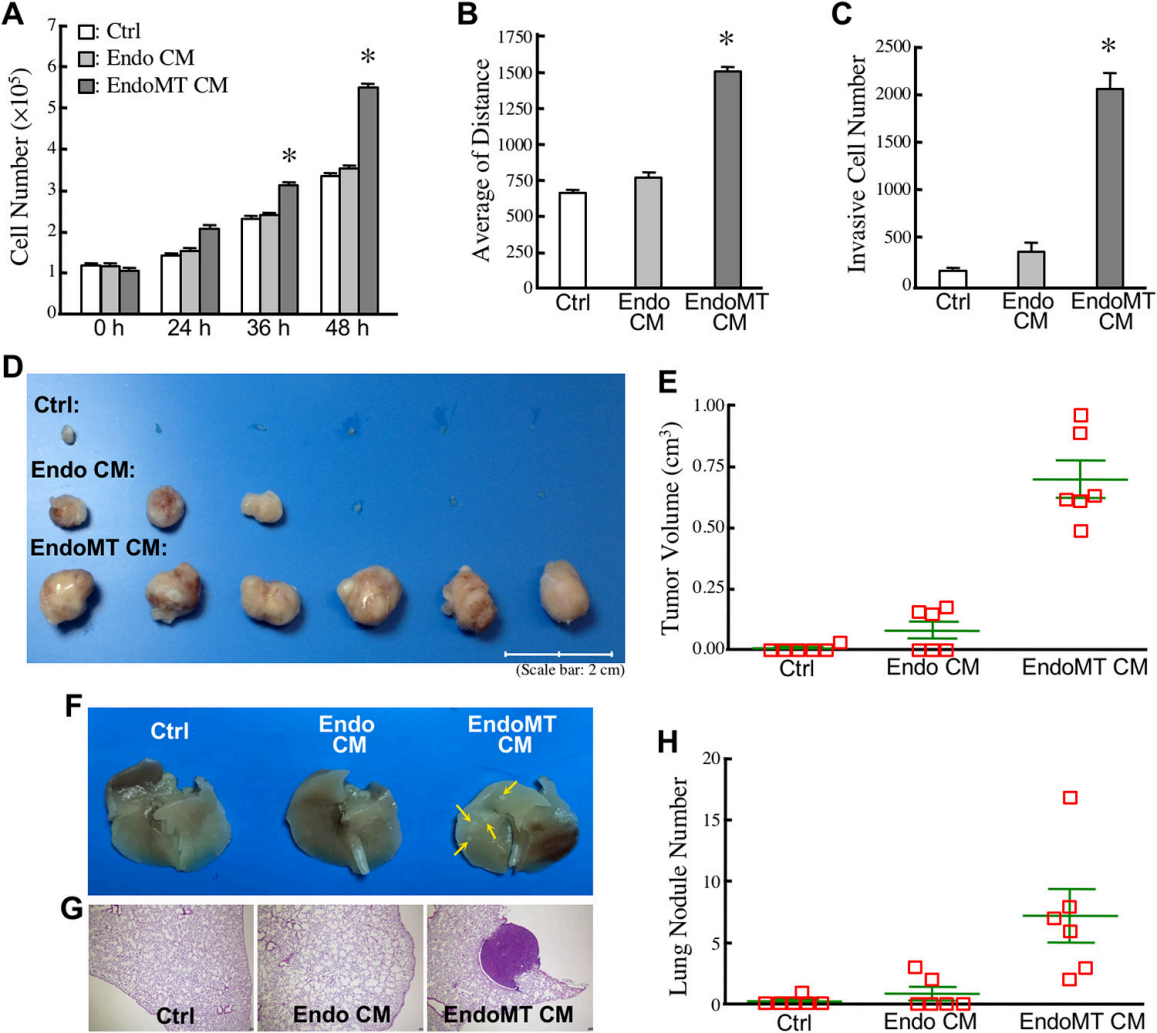
EndoMT enhances CRC malignancy **(A)** Cell proliferation levels of HCT-115 cells incubated with control medium (Ctrl), Endo CM, or EndoMT CM for 24, 36, and 48 h. The mean ± SD values of 3 independent experiments are shown. ^*^, *P* < 0.05 when compared with Ctrl. **(B)** Cell migration activities of HCT-115 cells incubated with control medium (Ctrl), Endo CM, or EndoMT CM. The mean ± SD values of 3 independent experiments are shown. ^*^, *P* < 0.05 when compared with Ctrl. **(C)** Invasiveness of HCT-115 cells stimulated with control medium (Ctrl), Endo CM, or EndoMT CM. The mean ± SD values of 3 independent experiments are shown. ^*^, *P* < 0.05 when compared with Ctrl. **(D)** Tumor formation levels of HCT-115 cells pre-incubated 24 h with control medium (Ctrl), Endo CM, or EndoMT CM. **(E)** Quantification of the tumor volumes as shown in (D). **(F)** Examples of the lungs from NOD-SCID mice intravenously inoculated with the HCT-115 cells pre-incubated 24 h with control medium (Ctrl), Endo CM, or EndoMT CM. Tumor nodules were indicated by arrows. **(G)** Example of the hematoxylin/eosin (H/E) staining result of the lung tissue sections to confirm the tumor mass. **(H)** Quantification of tumor nodules on the lungs from NOD-SCID mice intravenously inoculated with the HCT-115 cells pre-incubated 24 h with control medium (Ctrl), Endo CM, or EndoMT CM.

To investigate whether EndoMT-derived cells secrete certain proteins to stimulate CRC malignancy, we treated human CRC cell line HCT-115 with the control and conditioned media collected from HUVECs and EndoMT-derived cells, respectively. The activities of cell proliferation, migration, invasion, tumorigeneity, and lung colonization of HCT-115 cells were assayed after the treatment. Cellular proliferation levels were analyzed based on the numbers of viable HCT-115 cells counted after incubation for 24, 36, and 48 h (Figure 6A). Increased cell-proliferation rates were significantly observed with HCT-115 cells incubated with conditioned media of EndoMT cells (designated as “EndoMT CM”) but not with conditioned media of HUVECs (designated as “Endo CM”). By monitoring cell migration tracks using time-lapse photography, increased cell migration activity was also observed with HCT-115 cells incubated with EndoMT CM when compared with those with control medium or Endo CM (Figure 6B). As expected, the invasion ability of HCT-115 cells was also induced after incubated with EndoMT CM (Figure 6C). Furthermore, to assay the tumorigeneity and lung colonization activity, HCT-115 cells were incubated 24 h with different conditioned media and injected into NOD-SCID mice subcutaneously and intravenously, respectively. HCT-115 cells are a CRC cell line with low tumorigeneity and low metastatic activity. An incubation with control medium did not confer HCT-115 cell abilities to form tumor masses and lung nodules (Figure 6D-6H). However, Endo CM showed a stimulatory effect on HCT-115 cell tumorigeneity in 3 out of 6 mice, resulting in an average tumor volume of 0.12 cm^3^. EndoMT CM was even more potent to stimulate tumor growth in all 6 mice, with an increased average tumor volume up to 0.62 cm^3^ (Figure 6D, 6E). Additionally, the metastatic ability of HCT-115 cells was not significantly stimulated by Endo CM, thus producing an average number of 1 lung nodule in NOD-SCID mice. HCT-115 cells stimulated by EndoMT CM were shown to drastically reside in lungs, with an average number of lung nodule number to 7 (Figure 6F-6H).

### EndoMT produces eHSP90α to induce stemness in CRC cells

**Figure 7:**
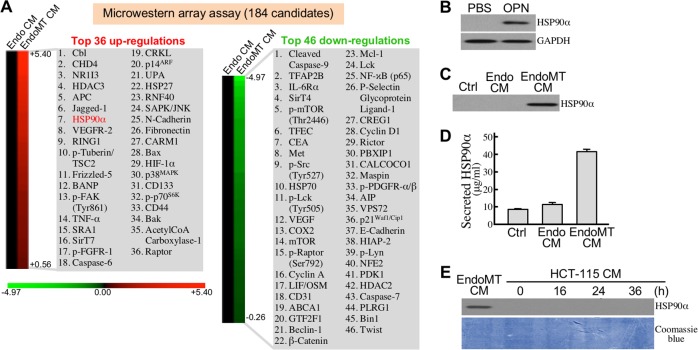
EndoMT-derived cells express and secrete HSP90α **(A)** List of top 36 up-regulated proteins and 46 down-regulated proteins secreted in EndoMT CM compared with Endo CM. **(B)** HSP90α levels in HUVECs treated 24 h with PBS or 0.3 μg/ml of OPN. **(C)** Detectable HSP90α level in 100-fold concentrated EndoMT CM. **(D)** HSP90α levels in control medium (Ctrl), Endo CM, and EndoMT CM, measured by enzyme-linked immunosorbent assay described in Materials and Methods. **(E)** No detectable level of HSP90α in the 100-fold concentrated media of HCT-115 cells treated with 2% FBS-containing M199 medium for 16, 24, or 36 h.

**Figure 8:**
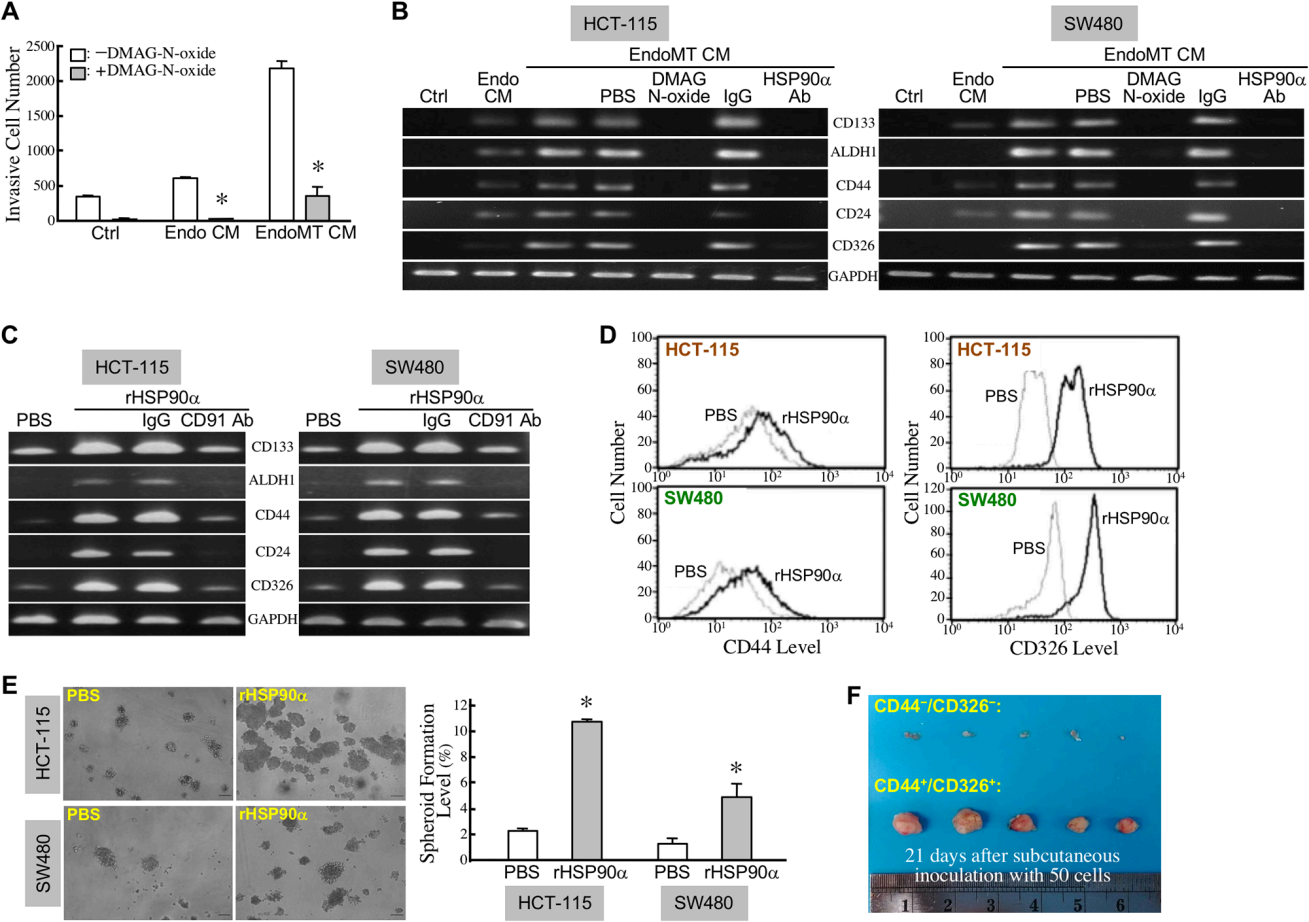
EndoMT produces eHSP90α to induce stemness in CRC cells **(A)** Invasiveness of the HCT-115 cells stimulated with control medium (Ctrl), Endo CM, or EndoMT CM in the absence or presence of 5 μM of DMAG-N-oxide. The mean ± SD values of 3 independent experiments are shown. ^*^, *P* < 0.05 when compared with the treatment in the absence of DMAG-N-oxide. **(B)** mRNA levels of CD133, ALDH1, CD44, CD24, and CD326 in the HCT-115 and SW480 cells treated 24 h with control medium (Ctrl), Endo CM, or EndoMT CM in the absence or presence of 5 μM of DMAG-N-oxide or 10 μg/ml of anti-HSP90α antibody. **(C)** mRNA levels of CD133, ALDH1, CD44, CD24, and CD326 in the HCT-115 and SW480 cells treated with PBS, 15 μg/ml rHSP90α, or 15 μg/ml rHSP90α plus 10 μg/ml control IgG or anti-CD91 antibody (AbD Serotec, Raleigh, NC, USA). **(D)** Flow cytometric analyses of cell-surface levels of CD44 and CD326 in the HCT-115 and SW480 cells treated with PBS or 15 μg/ml of rHSP90α. **(E)** Spheroid-forming abilities of PBS or rHSP90α-treated HCT-115 and SW480 cells in serum-free and anchorage-independent culture conditions. ^*^, *P* < 0.05 when compared with the data of PBS-treated cells. **(F)** Tumor-generating ability of 50 HCT-115 cells expressing CD326 and CD44. rHSP90α-treated HCT-115 cells were stained with fluorescence-labeled antibodies against CD326 and CD44, and sorted by flow cytometry. Fifty HCT-115 cells expressing CD44^+^/CD326^+^ were subcutaneously inoculated into each NOD-SCID mouse (n = 5). After 21 days, the tumor masses were surgically removed for observation.

To investigate which proteins were secreted in EndoMT CM and involved in the enhancement of CRC cell malignancy, we performed microwestern array assay on EndoMT CM *vs*. Endo CM using 184 antibodies (Supplementary Table 1). The top 36 up-regulated and top 46 down-regulated proteins in EndoMT CM were listed in Figure 7A. Among them, HSP90α was validated to be expressed and secreted more by EndoMT-derived cells (Figure 7B-7D). We previously reported that CRC cells secreted HSP90α upon serum-starvation stress. As shown in Figure 5E, no detectable level of HSP90α was found in the conditioned media of the HCT-115 cells treated with 2% FBS-containing M199 medium for 16, 24, or 36 h. Next, we investigated whether the secreted HSP90α in EndoMT CM was responsible for the promotion of CRC cell malignancy. Enhancement of HCT-115 cell invasiveness by EndoMT CM was significantly suppressed by the eHSP90α inhibitor, DMAG-N-oxide (Figure 8A). Given that development of cancer malignancy is usually accompanied by exhibition of stemness features; we therefore studied if the promotion of cancer malignancy by EndoMT CM could be attributed by eHSP90α-induced cancer cell stemness. Indeed, EndoMT CM induced gene expressions of stemness markers including CD133, ALDH1, CD44, CD24, and CD326 in HCT-115 and SW480 cells, which could be drastically antagonized by the presence of DMAG-N-oxide or anti-HSP90α antibody (Figure 8B). We further examined the effects of recombinant HSP90α (rHSP90α) on cellular stemness marker expression, spheroid formation, and tumorigeneity. The mRNA levels of CD133, ALDH1, CD44, CD24, and CD326 were all induced by rHSP90α in a CD91-dependent manner in HCT-115 and SW480 cells (Figure 8C). Consistently, cell-surface levels of CD44 and CD326 were elevated in these rHSP90α-treated CRC cells (Figure 8D). Cell-renewal activities, reflected by the spheroid-forming levels assayed in serum-free and non-adherent culture conditions, were also significantly increased by rHSP90α treatment when compared to control PBS treatment (Figure 8E). Additionally, the tumorigenicity was evaluated using 50 CD44^+^/CD326^+^ cells sorted from rHSP90α-treated HCT-115 cell population. As expected, all 5 NOD-SCID mice inoculated with those cells significantly developed tumor masses (Figure 8F). Taken together, EndoMT-derived cells secrete HSP90α and thus contribute to development of stemness in CRC cells.

## DISCUSSION

OPN is well reported to induce angiogenesis in human cancers [29, 30]. Angiogenesis needs EC proliferation and differentiation, and also involves EndoMT to render migration/invasion of developing vessels into peripheral tissues. In our present study, OPN shows EndoMT-inducing activity, which is consistent with the published results showing that OPN is able to induce angiogenesis. Additionally, EndoMT is thought to contribute to a considerable population of CAFs which facilitate malignant development and progression by secreting growth factors and extracellular matrix molecules [9]. Therefore, induction of EndoMT by OPN can at least partly account for the correlation of elevated OPN expression/secretion with tumor development and malignant progression. EndoMT is an important type of cell plasticity involved in OPN-induced malignancy.

Furthermore, we studied the underlying mechanisms of OPN-induced EndoMT. The PLA data confirmed that integrin α_V_β_3_ and CD44 act as endothelial cell receptors for OPN. However, OPN induces PI3K–Akt pathway through integrin α_V_β_3_ rather than CD44, resulting in an increased level of mTORC1-dependent HIF-1α protein expression. HIF-1α functions as a transcriptional factor to trans-activate *TCF12* gene expression. TCF12 further interacts with EZH2 and HDACs to transcriptionally repress *VE-cadherin* gene expression and thus facilitates EndoMT. We have previously identified TCF12 as a transcriptional repressor of *E-cadherin* gene and its overexpression promotes CRC cell EMT, migration, invasion, and metastasis [25]. TCF12 can be induced by eHSP90α in a NF-κB-mediated manner and is required for eHSP90α-stimulated CRC cell EMT, migration, and invasion [27]. In the present study, we report that TCF12 transcriptionally inhibits *VE-cadherin* gene expression and is involved in OPN-induced EndoMT. OPN is generally overexpressed in many cancers including CRC and associated with the malignant progression [29, 31]. Disclosing the TCF12 signaling axis in OPN-induced EndoMT may shed light on the unknown mechanism of cancer-related EndoMT. Moreover, our data demonstrate that HIF-1α binds to *TCF12* gene promoter region and up-regulates TCF12 expression. HIF-1α has been known as an important regulatory factor in cell EMT, migration, invasion, angiogenesis, metabolism, and autophagy [18, 32]. Our present study further suggests that HIF-1α promotes EndoMT through TCF12 induction.

HIF-1α, forming a heterodimer with the constitutively expressed HIF-1β, can mediate many cellular adaptive responses [18]. HIF-1α is hydroxylated and susceptible to proteasomal degradation under normoxia. Besides hydroxylation, other posttranslational modifications including sumoylation, acetylation, phosphorylation, and methylation are also known to regulate HIF-1α stability. Sumoylation and acetylation of HIF-1α facilitates VHL-mediated ubiquitination and degradation of HIF-1α [33, 34], whereas phosphorylation by p38^MAPK^ prevents HIF-1α from interacting with VHL [35]. HIF-1α can be methylated by SET7/9 methyltransferase in nucleus under normoxia and long-term hypoxia, while lysine-specific histone demethylase 1 (LSD1) can reverse this methylation and lead to HIF-1α stabilization under hypoxic conditions [36]. LSD1 can also demethylate RACK1 and hence disrupt RACK1–HIF-1α interaction [37]. Unlike VHL, RACK1 can trigger ubiquitination and proteasomal degradation of HIF-1α independent of oxygen. Complementing the regulation of HIF-1α stability, PI3K activation stimulates Akt and mTORC1-mediated HIF-1α translation [20, 21]. Our study revealed that OPN induced protein but not mRNA level of HIF-1α during EndoMT. The OPN-induced HIF-1α protein level was drastically abolished by rapamycin, suggesting that the protein was induced through an mTORC1-dependent *de novo* synthesis mechanism. TSC2 and AMP-activated protein kinase (AMPK) are two Akt-regulated mTORC1 suppressors [21, 38]. Inactivating phosphorylation of TSC2 but not AMPK was induced in OPN-treated ECs. Together, these data suggest that OPN induces TCF12-mediated EndoMT through a PI3K–Akt–TSC2–mTORC1–HIF-1α signaling axis.

To study the significance of EndoMT in cancer development and progression, we analyzed the proteins secreted by EndoMT-derived cells through microwestern array assay. We have noticed that the levels of Jagged-1 and HSP90α were increased by approximately 3.2-fold in EndoMT CM compared with Endo CM. Jagged-1 is originally identified as a membrane-bound ligand that activates Notch signaling in contacting cells [39]. After the proteolytic cleavage by ADAM17, N-terminal soluble form of Jagged-1 is produced by ECs to bind and activate the receptor Notch on CRC cells to promote cancer stemness [40]. This indicates that ECs are not only involved in angiogenesis in tumor microenvironment, but also contribute to cancer development and progression by releasing factors like Jagged-1. In our study, EndoMT CM was more potent than Endo CM to promote the tumor growth and metastasis of CRC cell xenografts in NOD-SCID mice. Indeed, EndoMT-derived cells produced more soluble Jagged-1 and eHSP90α than ECs did. HSP90α is a well-known chaperone responsible for cellular folding, maturation, and trafficking of many client oncoproteins such as HIF-1α, Akt, mutated p53, ErbB2/Neu, and Bcr-Abl [41, 42]. However, HSP90α is not restricted to cytoplasmic localization. It can be produced and secreted from the fibroblasts in damaged tissues, as well as from cancer cells [43–47]. Clinically, elevated levels of serum/plasma HSP90α are associated with malignant progression in CRC, non-small cell lung cancer, breast carcinoma, pancreatic ductal adenocarcinoma, hepatocellular carcinoma, and glioblastoma [44–47]. CD91 is known as a receptor for eHSP90α on CRC cells [46]; through CD91, eHSP90α induces NF-κB-mediated TCF12 expression to promote CRC cell EMT, migration, and invasion [27]. Recently, eHSP90α is reported to induce stemness in prostate cancer cells [48]. Our data also revealed that rHSP90α alone was able to induce CRC cell stemness, and induction of CRC cell invasion and stemness by EndoMT CM was efficiently suppressed by anti-HSP90α antibody and eHSP90α inhibitor, DMAG-N-oxide. Our study suggests that the cancer-promoting effects of EndoMT can be at least partly attributed by the production of eHSP90α and soluble Jagged-1.

In summary, our data demonstrate that the PI3K–Akt–TSC2–mTORC1–HIF-1α–TCF12 signaling cascade is responsible for OPN-induced EndoMT. eHSP90α and soluble Jagged-1 are also revealed as the two underlying factors contributing to the cancer-promoting effects of EndoMT. Like CAFs, EndoMT-derived cells and even the process of EndoMT can be potentially taken as the therapeutic targets. Considering OPN overexpression is ubiquitous in human cancers and HIF-1α is one of the most important transcriptional factors for malignant transformation of tumor cells, the OPN-induced and HIF-1α-mediated signaling cascade can be reasonably targeted for the prevention of OPN-induced EndoMT to inhibit cancer development and progression. To address PI3K/Akt/mTORC1 signaling, the dual PI3K/mTOR inhibitors NVP-BEZ235, GSK2126458, and XL765 as well as the newest generation of mTOR inhibitor RapaLink-1 have been developed and exhibit their superiorities over the early generation of mTOR inhibitors like rapamycin (sirolimus), RAD001 (everolimus), and CCI-779 (temsirolimus) [49]. Several inhibitors targeting HIF-1α pathway have also been developed for cancer therapeutics [50]. Among them, tanespimycin (17-N-allylamino-17-demethoxygeldanamycin, 17-AAG), a potent HSP90 inhibitor, exhibits suppressive effect on HSP90 binding to HIF-1α and eventually leads to acceleration of HIF-1α degradation by RACK1. On the other hand, blocking Jagged-1-Notch signaling in combination with conventional chemotherapy has been considered as an attracting strategy to treat several malignant cancers [51]. Moreover, reagents targeting eHSP90a like DMAG-N-oxide and anti-HSP90a antibodies have also been developing in recent years and the promising efficacies have been shown in mouse models [52, 53].

## MATERIALS AND METHODS

### Clinical CRC specimens

CRC tissue sections were obtained from Taipei Veterans General Hospital with written informed consent from 15 patients, according to the medical ethics protocol approved by the Human Clinical Trial Committee of Taipei Veterans General Hospital.

### Antibodies

For immunohistofluorescence, anti-α-SMA mouse monoclonal antibody (sc-32251, 1:50), anti-CD31 goat polyclonal antibody (sc-1505, 1:50), and anti-OPN mouse monoclonal antibody (sc-21742, 1:50) were purchased from Santa Cruz Biotechnology (Santa Cruz, CA, USA). Anti-CD68 rabbit polyclonal antibody (#76437, 1:200) was from Cell Signaling (Danvers, MA, USA). For immunoblotting, anti-Tie1 (GTX107818, 1:1000), anti-Tie2 (GTX107505, 1:1000), and anti-HSP90α (GTX109753, 1:1000) antibodies were obtained from GeneTex Inc. (Hsinchu City, Taiwan). Anti-CD31 (sc-1505, 1:500), anti-VE-cadherin (sc-6458, 1:500), anti-α-SMA (sc-32251, 1:500), anti-fibronectin (sc-59824, 1:500), anti-TCF12 (sc-28364, 1:500), anti-Twist-1 (sc-15393, 1:500), anti-Snail (sc-10432, 1:500), anti-HDAC1 (sc-81598, 1:500), anti-HDAC2 (sc-6296, 1:500), anti-HDAC3 (sc-17995, 1:500), and anti-Akt (sc-1619, 1:500) antibodies were from Santa Cruz Biotechnology. Anti-Bmi1 antibody (#05-637, 1:1000) was from EMD Millipore (Billerica, MA, USA). Anti-EZH2 (#3147, 1:1000), anti-phospho-Ser-473-Akt (#9271, 1:1000), anti-phospho-Thr-1462-TSC2 (#3611, 1:1000), anti-TSC2 (#4308, 1:1000), anti-phospho-Thr-172-AMPK (#2532, 1:1000), anti-AMPK (#2535, 1:1000), anti-phospho-Ser-2448-mTOR (#5536, 1:1000), anti-mTOR (#2983, 1:1000), anti-phospho-Thr-389-p70^S6K^ (#9205, 1:1000), anti-p70^S6K^ (#2708, 1:1000), anti-phospho-Ser-65-4E-BP1 (#9456, 1:1000), and anti-4E-BP1 (#9644, 1:1000) antibodies were from Cell Signaling. Anti-GAPDH antibody (NB300-221, 1:100000) was from Novus Biologicals (Littleton, CO, USA). Anti-HIF-1α antibody (#610959, 1:1000) was from BD Biosciences (San Jose, CA, USA). For ChIP, anti-TCF12 and anti-HIF-1α antibodies were same as above. Anti-H3K27me3 (GTX54106, 1:1000) and anti-H3K27ac (GTX128944, 1:1000) antibodies were from GeneTex Inc.

### Immunohistofluorescence

Tissue sections were deparaffinized by xylene and then rehydrated by a series of ethanol dilutions. Antigen retrieval was carried out by heating 15 min in 10 mM citrate buffer, pH 6.0. The tissue sections were then blocked in 3% BSA in PBS for 30 min prior to overnight incubation with primary antibodies. After PBS washes, anti-mouse IgG-Alexa Fluor 488, anti-rabbit IgG-Alexa Fluor 568, and anti-goat IgG-Alexa Fluor 647 secondary antibodies (1:500; Thermo Fisher Scientific, Waltham, MA, USA) were applied and nuclei were stained with 4’,6’-diamidino-2-phenylindole (DAPI). Results were observed and photographed under Leica TCS SP5 II confocal microscope and LASAF software (Leica, Wetzlar, Germany).

### Cell culture

Human umbilical vein endothelial cells (HUVECs) were isolated from umbilical cords of normal deliveries according to the procedure described previously [54] and approved by the Human Clinical Trial Committee of Taipei Veterans General Hospital. HUVECs were cultivated in M199 medium (Thermo Fisher Scientific) plus 20% of fetal bovine serum (FBS), 100 units/ml of penicillin, 100 μg/ml of streptomycin, and 30 μg/ml of endothelial cell growth supplement and incubated at 37°C, 5% CO_2_. Human immortalized EC line EC-RF24 (kindly provided by Dr. Lee M. Ellis [40]) and human CRC cell lines HCT-115 and SW480 were grown in RPMI 1640 medium supplemented with 10% FBS, 100 units/ml of penicillin, 100 μg/ml of streptomycin, and 2 mM of L-glutamine.

### TCF12 knockdown and overexpression in ECs

To generate TCF12-knockdown ECs, HUVECs were infected with pLKO.1 puro plasmids carrying TCF12 shRNA-expressing sequence 5’-CCC-ACA-ATT-CTT-CTG-ACC-TTT-3’ (#169) or 5’-GCA-ATC-ATT-CAG-TCC-TGT-CTA-3’ (#172) which were provided by National RNAi Core Facility (Taipei, Taiwan). To generate TCF12-overexpressing ECs, EC-RF24 cells were transfected with pcDNA3-HA plasmid carrying TCF12 cDNA (10 μg of plasmid DNA per 2×10^6^ cells for 48 h) using TurboFect transfection reagents (Thermo Fisher Scientific).

### RNA isolation and RT-PCR

Cellular RNA was isolated using TRIzol reagent (Thermo Fisher Scientific), and the mRNA included was converted to cDNA by Tetro Reverse Transcriptase (Bioline Reagents Ltd., London, UK). The cDNA products were further added with Hot-start DNA polymerase (Thermo Fisher Scientific) to proceed PCR reactions. Real-time quantitative PCR (qPCR) was performed with QuantiNova SYBR Green RT-PCR Kit (Qiagen, Hilden, Germany) in a RotorGene 3000 system (Corbett Research, Mortlake, Australia). The primer sequences and reaction conditions were listed in Supplementary Table 2.

### Cell lysis for immunoblot analysis

Cell lysates were prepared by briefly sonicating cells in modified RIPA buffer (10 mM Na_2_HPO_4_, 1.8 mM KH_2_PO_4_, 137 mM NaCl, 2.7 mM KCl, 0.5% deoxycholate, 1% Nonidet P-40, 0.3% SDS) plus cocktails of protease inhibitors and phosphatase inhibitors (Sigma, St. Louis, MO, USA). After protein concentrations were determined by BCA protein assay kit (Thermo Fisher Scientific), cell lysates were subjected to conventional SDS-PAGE and immunoblot analysis procedures. Finally, protein bands were detected by enhanced chemiluminescence (Luminata^™^ Crescendo Western HRP Substrate, EMD Millipore).

### Gap-junction activity assay

Calcein-transfer assay was performed to evaluate EC gap-junction activities [25]. PBS or OPN-treated ECs (HUVECs or EC-RF-24 cells) were trypsinized and labeled with Calcein acetoxymethyl ester and 1,1-dioctadecyl-3,3,3,3-tetramethylindocarbocyanine perchlorate (DiI) dyes, and then co-cultured with unlabeled, untreated EC monolayer for 15 min or 3 h. After PBS wash, the monolayer cells were trypsinized for flow cytometric analyses.

### Cell migration assay

For assaying the effect of OPN on EC migration, HUVECs or EC-RF-24 cells (2×10^6^ cells/10-cm dish) were pre-incubated with 2% FBS-containing medium for 16 h. PBS or 0.3 μg/ml OPN was added with/without the antibody against integrin α_V_β_3_ (#23C6, R&D Systems, Minneapolis, MN, USA) or CD44 (sc-72110, Santa Cruz Biotechnology) or the inhibitor targeting PI3K (LY294002, Cell Signaling) or IKKα/β (Merck, Darmstadt, Germany) for another 24 h. After cells were wounded with white tips, washed twice with PBS, and incubated with fresh treating media, images of cells migrating into the wounded area were taken every 10 min for 15 h using CCM-330F system (Astec Co., Fukuoka, Japan) and migration distances were quantified using Image-Pro Plus Version 5.0.2 software (MediaCybernetics Inc., Silver Spring, MD, USA). For assaying the effect of EndoMT on CRC cell migration, HCT-115 cells were pre-incubated with 1% FBS-containing medium for 16 h, and incubated with HUVECs or EndoMT cells-conditioned media for another 24 h.

### Transwell invasion assay

ECs and HCT-115 cells were treated as described above. Treated cells were trypsinized, counted, suspended in the fresh treating media, and then seeded into the top chambers of the Transwell inserts pre-coated with 5-fold diluted Matrigel. The cells were allowed 15 h to invade through the Matrigel. Invasive cells on the filters of Transwell inserts were staining with Giemsa dye and counted using Image-Pro Plus software.

### Chromatin immunoprecipitation (ChIP) assay

ChIP assay was performed on PBS or OPN-treated HUVECs according to the manufacturer's instruction of EZ-ChIP kit (EMD Millipore). Cells were treated with 1% formaldehyde for cross-linking, followed by lysate preparation and DNA fragmentation. After preclearing with protein G-conjugated agarose, 10-μl aliquots of cell lysates were saved as “input” fractions and the remaining lysates were added with control IgG or interested antibodies for immunoprecipitation. Furthermore, DNA was extracted from the immunoprecipitates for PCR using the primers and conditions as follows: the E-box-containing region of *VE-cadherin* gene promoter [forward, 5′-ATC-CCA-TCC-AGC-ACC-TTG-TA-3′; reverse, 5′-GTG-ACC-TTG-GCC-ATT-AGC-AT-3′; 95°C (30 sec), 50°C (30 sec), and 72°C (30 sec) for 35 cycles], the HRE site-1 (-440 to -435)-containing region of *TCF12* gene promoter [forward, 5′-TCT-GCG-CCG-ACT-GCA-GCC-CT-3′; reverse, 5′-ATC-CGT-GCC-CAG-GTC-CCC-GA-3′; 95°C (30 sec), 62°C (45 sec), and 72°C (45 sec) for 35 cycles], and HRE site-2 (-728 to -723)-containing region of *TCF12* gene promoter [forward, 5′-GGG-CTG-TCT-CCG-TTA-GAT-GA-3′; reverse, 5′-AGA-CCC-CAC-ATC-TCC-AGT-CA-3′; 95°C (30 sec), 54°C (45 sec), and 72°C (45 sec) for 35 cycles].

### Nuclear extraction for immunoprecipitation

Nuclear proteins of PBS or OPN-treated HUVECs were isolated according to the procedure described previously [25]. Aliquots (500 μg) of nuclear extracts were incubated overnight at 4°C with 10 μg of control IgG or anti-TCF12 antibody (sc-28364, 1:500, Santa Cruz Biotechnology). After incubation with protein A/G-Sepharose for another 2 h, the immunoprecipitates were washed and resolved by 12% SDS-PAGE and analyzed by immunoblotting with interested antibodies.

### E-box pull-down and immunoblot analysis

Based on ChIP assay, we synthesized a pair of biotin-conjugated oligonucleotides containing the E-box site of *VE-cadherin* gene promoter. The sequences were: forward, 5′-GAC-TGG-GCT-CAC-CCC-AGA-TCA-GCT-GAT-TTG-GAA-TCT-CCC-3′; reverse, 5′- GGG-AGA-TTC-CAA-ATC-AGC-TGA-TCT-GGG-GTG-AGC-CCA-GTC-3′. The oligonucleotides were incubated 16 h at 4°C with 300 μg of nuclear extracts isolated from PBS or OPN-treated HUVECs. The reaction mixtures were then added with 20 μl of Streptavidin Mag Sepharose (GE Healthcare, Piscataway, NJ, USA) and incubated on a rotatory shaker for another 3 h. E-box-associated proteins were pulled down after centrifugation at 3000×*g* for 5 min at 4°C and subjected to immunoblot analyses.

### *In silico* analyses of the microarray datasets of clinical specimens

The relationships between the mRNA expression levels of two genes in CRC tissue specimens were investigated through the meta-analyses of cDNA microarray datasets deposited in the website “http://www.oncomine.com”. The significance of the correlations between two genes was evaluated by the Pearson's product-moment correlation coefficients calculated using SPSS 11.0 software (SPSS Inc., Chicago, IL, USA).

### Proximity ligation assay

HUVECs, seeded on glass coverslips (2×10^5^ cells per 12-mmØ coverslip) were pre-incubated with 2% FBS-containing medium for 16 h and treated with PBS or 0.3 μg/ml of OPN for another 45 min. Treated cells were fixed with 3% paraformaldehyde and blocked with the blocking solution supplied in the Duolink *in situ* PLA kit (Olink Bioscience, Uppsala, Sweden). Cells were then incubated with 4 μg/ml of anti-CD44 antibody (sc-72110, Santa Cruz Biotechnology) or anti-integrin α_V_β_3_ antibody (#23C6, R&D Systems) at 4°C for 45 min. After washed thrice with Tris-buffered saline plus 0.05% Tween 20, cells were further incubated with 6.25 μg/ml of anti-OPN antibody (sc-21742, Santa Cruz Biotechnology) at 4°C for overnight. The subsequent procedure for detecting physical associations of OPN with integrin α_V_β_3_ and CD44 was performed as described in the manufacturer's instructions of the Duolink *in situ* PLA kit. Nuclei were counterstained with DAPI for 2 min in the dark at room temperature. Coverslips were mounted with mounting solution overnight in the dark at room temperature, and the results were observed and photographed using the Leica TCS SP5 II confocal microscope and LASAF software.

### Preparation of Endo and EndoMT conditioned media

HUVECs (2×10^6^ cells per 10-cm dish) were pre-incubated 16 h with 2% FBS-containing medium and treated 24 h with PBS or 0.3 μg/ml of OPN in 2% FBS-containing medium. After PBS wash, the control or OPN-treated HUVECs were incubated with 5 ml of fresh 2% FBS-containing medium for another 24 h. A dish containing the medium without cells was set in parallel as the control medium. All media were collected and subjected to 0.22-μm filtration for experiments (designated as “Ctrl”, “Endo CM”, and “EndoMT CM”, respectively).

### Cell proliferation

HCT-115 cells (1×10^5^ cells per 10-cm dish) were pre-incubated with 1% FBS-containing medium for 16 h, and then incubated with control medium, Endo CM, or EndoMT CM for another 24, 36, and 48 h. Treated cells were trypsinized, suspended in PBS, and added with Trypan blue dye. The cells excluding Trypan blue were counted as living cells.

### Microwestern array assay

Endo CM and EndoMT CM (each 50 ml) were concentrated by 100 folds using Vivaspin centrifugal concentrators, and then arrayed on SDS-polyacrylamide gels for microwestern array assay performed by the Micro-Western Array core facility of National Health Research Institutes. After resolved by semidry electrophoreses, samples were transferred onto nitrocellulose membranes and probed with 184 antibodies.

### Measurement of secreted HSP90α levels

Control medium, Endo CM, and EndoMT CM were loaded into 96-well plates (100 μl per well). To each well, 1 μg/ml of anti-HSP90α antibody (AbD Serotec, Raleigh, NC, USA) was added and incubated at 37°C for 1 h. After three washes, horseradish peroxidase-conjugated secondary antibody was added and incubated at 37°C for another hour. The substrate 3,3’,5,5’-tetramethylbenzidine in 0.015% H_2_O_2_ was added and the reaction proceeded at room temperature in the dark for 10 min. Finally, the reactions were stopped by 0.5 M H_2_SO_4_ and the OD was detected at 450 nm using Infinite M200 microplate reader (TECAN, Männedorf, Switzerland). A series of concentrations of rHSP90α (StressGen Inc., Ann Arbor, MI, USA) was prepared in 0.05 mg/ml BSA to be used as standards.

### Flow cytometric analyses of cell-surface stemness markers

PBS or rHSP90-treated HCT-115 or SW480 cells were trypsinized and collected, 1×10^5^ cells in 50 μl of ice-cold PBS plus 1% FBS were added with Alexa Fluor 488-conjugated anti-CD44 antibody or phycoerythrin-conjugated anti-CD326 antibody at the concentrations suggested by the manufacturer (BioLegend, San Diego, CA, USA). After 1-h incubation at room temperature and three times 20-min washes with PBS plus 1% FBS, the cells were resuspended in PBS plus 1% FBS and analyzed immediately by FACSCalibur flow cytometer (BD Biosciences).

### Cell-spheroid formation assay

PBS or rHSP90-treated HCT-115 or SW480 cells were trypsinized, suspended in serum-free medium, and seeded onto agar-coating 24-well dishes (1×10^3^ cells per well). The cells were incubated for 14 days, and the serum-free medium was refreshed every 3 days. Finally, cell spheroids (>100 μm in diameter) were stained with Giemsa dye and counted under an Olympus IX 71 inverted microscope (Center Valley, PA, USA). The level of cell spheroid formation was calculated as 100% × (number of cell spheroids/1000).

### Mouse tumor transplantation models

All mouse experiments were performed in accordance with the protocols approved by the Institutional Animal Care and Use Committee of National Health Research Institutes. HCT-115 cells were pre-incubated with 1% FBS-containing medium for 16 h, and incubated with control medium, Endo CM, or EndoMT CM for another 24 h. For tumor growth, treated HCT-115 cells were harvested in fresh treating media (5×10^5^ cells in 50 μl for each injection), mixed with an equal volume of Matrigel, and then subcutaneously injected into NOD-SCID mice (provided by National Laboratory Animal Center, Taipei, Taiwan). The mice were sacrificed and the tumor masses were surgically removed on Day-29 post-inoculation. The tumor volumes were calculated as 1/2 × length × width^2^ (cm^3^). For metastasis, treated HCT-115 cells (1×10^6^ cells in 100 μl PBS) were transplanted into NOD-SCID mice through tail-vein injections. On Day-41 post-inoculation, the mice were sacrificed for examination of tumor nodule formation on lungs. In addition, we assayed the tumorigeneity of 50 HCT-115 cells expressing CD326 and CD44. HCT-115 cells were treated 24 h with 15 μg/ml of rHSP90α, and then stained with fluorescence-labeled antibodies against CD326 and CD44 and sorted by flow cytometry. NOD-SCID mice were subcutaneously inoculated with 50 CD44^+^/CD326^+^ HCT-115 cells per mouse. After 21 days, the tumor masses were surgically removed for analysis.

## SUPPLEMENTARY MATERIALS FIGURES AND TABLES




