# Supplementary material for: Osteopontin–integrin engagement induces HIF-1α–TCF12-mediated endothelial-mesenchymal transition to exacerbate colorectal cancer
Source: Oncotarget. 2017 Dec 22;9(4):4998–5015. doi: 10.18632/oncotarget.23578 (PMC5797029; doi:10.18632/oncotarget.23578)
Supplement: Supplementary file 1 [file oncotarget-09-4998-s001.pdf]

Osteopontin–integrin engagement induces HIF-1α–TCF12-mediated endothelial-mesenchymal transition to exacerbate colorectal cancer

SUPPLEMENTARY MATERIALS

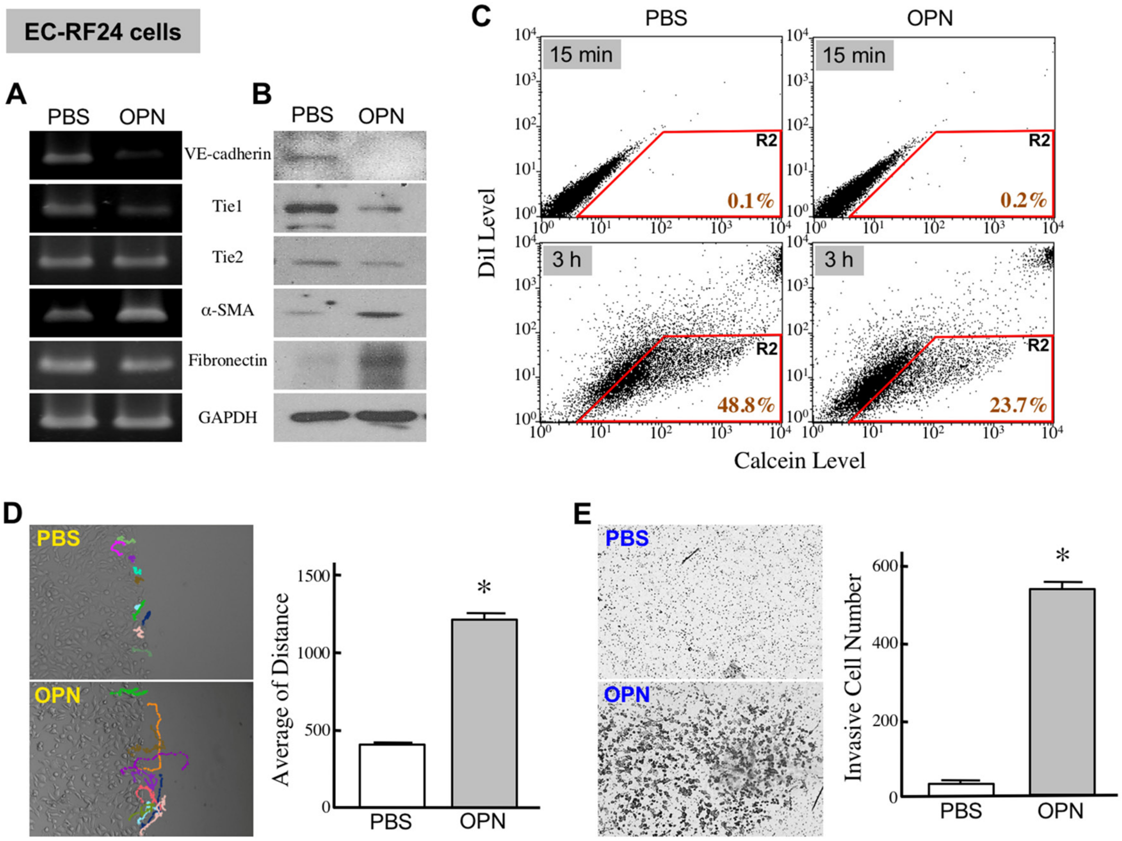

Supplementary Figure 1: OPN induces EndoMT in EC-RF-24 cells, an immortalized EC line. **(A)** mRNA levels of VE-cadherin, Tie1, Tie2, α-SMA, and fibronectin in EC-RF-24 cells pre-incubated 16 h with 2% FBS-containing medium and then treated with PBS or 0.3 µg/ml of OPN for another 15 h. **(B)** VE-cadherin, Tie1, Tie2, α-SMA, and fibronectin levels in EC-RF-24 cells pre-incubated 16 h with 2% FBS-containing medium and then treated 24 h with PBS or 0.3 µg/ml of OPN. It was noted that OPN changed the protein levels of Tie2 and fibronectin but did not significantly affect their mRNA levels. **(C)** Gap-junction activities in EC-RF24 cells treated with PBS or OPN as described in (B). Representative dot plots from 3 independent Calcein-transfer assays are shown. The cells in the R2 regions were categorized as Calcein-accepting cells, and the % of Calcein-accepting cells represented cellular gap-junction activities of the tested cells. **(D)** Cell migration activities in EC-RF-24 cells treated with PBS or OPN as described in Methods. Cell migration was monitored for 15 h using time-lapse photography, and the movement tracks of 10 randomly selected PBS or OPN-treated EC-RF-24 cells were analyzed by Image-Pro Plus software. Quantification of the accumulated migration distances is shown in the right panel. The mean ± SD values of 3 independent experiments are shown. *, $P < 0.05$ when compared with PBS-treated cells. **(E)** Invasiveness of PBS or OPN-treated EC-RF-24 cells determined by Transwell invasion assay. The representative images shown were invasive cells on the filters of Transwell inserts. The mean ± SD values of 3 independent experiments are shown in the right panel. *, $P < 0.05$ when compared with PBS-treated cells.

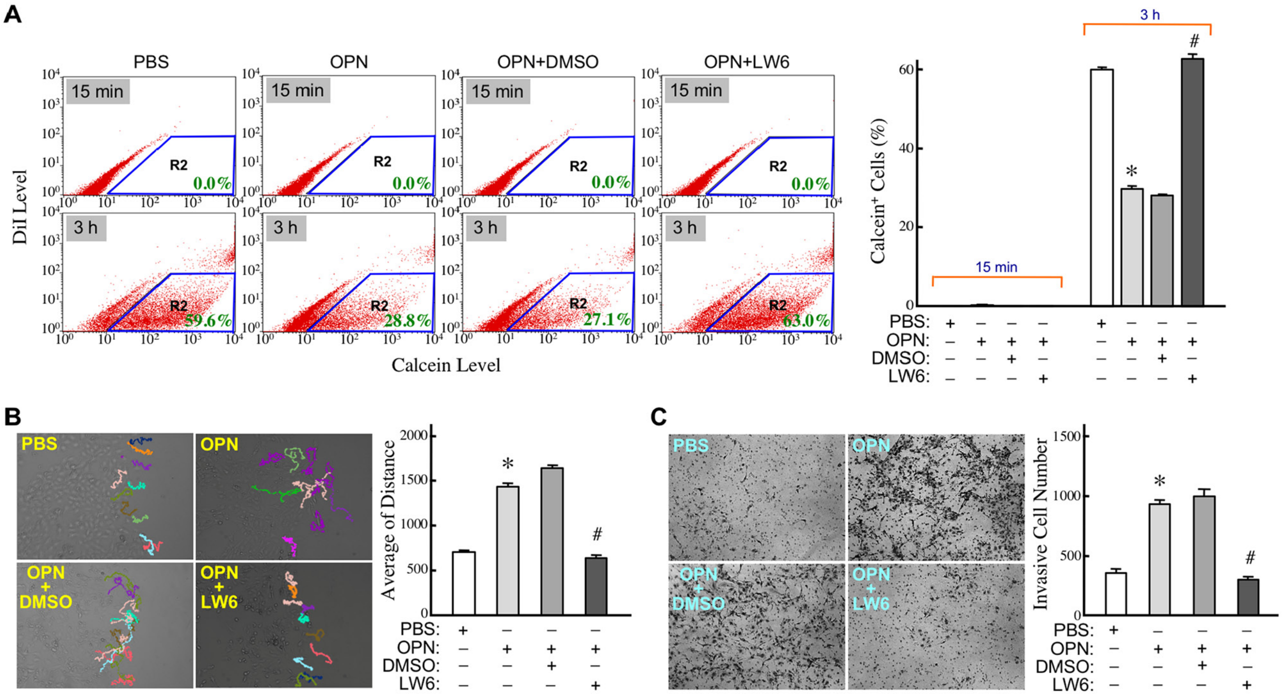

Supplementary Figure 2: Antagonistic effect of LW6 on OPN-inhibited cellular gap-junction as well as OPN-induced cell migration and invasion in HUNVECs. Assays of cellular gap-junction **(A)**, migration **(B)**, and invasion **(C)** activities in HUVECs treated as described previously with PBS, OPN, or OPN together with DMSO or LW6. In each panel, the left side is the representative plots/images, and the quantitative data shown in the right side are mean ± SD values of 3 independent experiments. *, $P < 0.05$ when compared with PBS-treated cells. #, $P < 0.05$ when compared with the treatment with OPN plus DMSO.

Supplementary Table 1: Microwestern array assay of protein levels in Endo CM *vs*. EndoMT CM

See Supplementary File 1

Supplementary Table 2: RT-PCR analyses of mRNA levels of the following genes. The primers and PCR conditions were listed below. All reactions started at 95°C for 5 min and terminated at 72°C for 7 min

Gene	Primer Sequence	PCR Condition & Product Size
VE-cadherin	Forward: 5′-GTT-TCG-TGG-TGT-TAT-GTC-CT-3′<br>Reverse: 5′-AGT-TGT-TCC-GAG-TCA-CAA-AA-3′	95°C (30 sec), 49°C (40 sec), and<br>72°C (40 sec) for 32 cycles; 243 bp
Tie1	Forward: 5′-AGT-TTC-GAG-GCT-GCT-CCA-3′<br>Reverse: 5′-TGT-GCT-GGT-CGG-AGA-GAA-3′	95°C (30 sec), 53°C (40 sec), and<br>72°C (40 sec) for 25 cycles; 282 bp
Tie2	Forward: 5′-GGC-AAG-AAG-GAA-CAG-CAG-A-3′<br>Reverse: 5′-GCA-AAA-GCA-GCA-GCA-GAA-G-3′	95°C (30 sec), 53°C (40 sec), and<br>72°C (40 sec) for 25 cycles; 267 bp
CD31	Forward: 5′-TGT-CTC-CAG-ACA-CCA-TTC-CA-3′<br>Reverse: 5′-TTA-GAG-CGC-CCT-CTT-GTG-TT-3′	95°C (30 sec), 52°C (45 sec), and<br>72°C (45 sec) for 25 cycles; 202 bp
α-SMA	Forward: 5′-TCC-AGA-GGC-ATA-GAG-AGA-CA-3′<br>Reverse: 5′-ACC-CTG-AAG-TAC-CCG-ATA-GA-3′	95°C (30 sec), 52°C (40 sec), and<br>72°C (40 sec) for 30 cycles; 222 bp
Fibronectin	Forward: 5′-ACC-AAC-CTA-CGG-ATG-ACT-CG-3′<br>Reverse, 5′-GCT-CAT-CAT-CTG-GCC-ATT-TT-3′	95°C (30 sec), 56°C (40 sec), and<br>72°C (40 sec) for 33 cycles; 229 bp
TCF12	Forward: 5′-GTC-GAT-TAG-GAG-CCC-ATG-AA-3′<br>Reverse: 5′-TAT-GCT-GTC-CCA-GGT-TTT-CC-3′	95°C (30 sec), 56°C (40 sec), and<br>72°C (40 sec) for 25 cycles; 202 bp
Twist-1	Forward: 5′-GGC-CGG-AGA-CCT-AGG-TAA-GG-3′<br>Reverse: 5′-CAT-CCC-CCT-GGA-GAC-TGC-3′	95°C (30 sec), 58°C (40 sec), and<br>72°C (40 sec) for 30 cycles; 218 bp
Snail	Forward: 5′-CAG-ACC-CAC-TCA-GAT-GTC-AA-3′<br>Reverse: 5′-CAT-AGT-TAG-TCA-CAC-CTC-GT-3′	95°C (45 sec), 53°C (45 sec), and<br>72°C (45 sec) for 30 cycles; 557 bp
HIF-1α	Forward: 5′-CAG-CTA-TTT-GCG-TGT-GAG-GA-3′<br>Reverse: 5′-CCA-AGC-AGG-TCA-TAG-GTG-GT-3′	95°C (30 sec), 62°C (1 min), and<br>72°C (1 min) for 30 cycles; 470 bp
CD133	Forward: 5′-ATC-CAT-CCT-ATC-CTG-GAA-GA-3′<br>Reverse: 5′-CCT-GTC-TGT-CTC-TTG-CAC-TC-3′	95°C (30 sec), 56°C (30 sec), and<br>72°C (1 min) for 30 cycles; 264 bp
ALDH1	Forward: 5′-CAA-GCA-GAC-ATG-ACA-TCC-TAG-G-3′<br>Reverse: 5′-TCC-TTT-GTA-GCC-TAC-TTT-TGC-A-3′	95°C (30 sec), 56°C (30 sec), and<br>72°C (1 min) for 30 cycles; 189 bp
CD44	Forward: 5′-GCA-GAA-TGT-GGA-CAT-GAA-GA-3′<br>Reverse: 5′-ATG-CTA-AAA-AAG-ATT-CGC-AAT-G-3′	95°C (30 sec), 56°C (30 sec), and<br>72°C (1 min) for 30 cycles; 150 bp
CD24	Forward: 5′-GGT-GCC-TGT-AAT-CCC-AGC-TA-3′<br>Reverse: 5′-CTC-CTG-AGG-CTT-TGG-ATT-TG-3′	95°C (30 sec), 56°C (30 sec), and<br>72°C (1 min) for 30 cycles; 394 bp
CD326	Forward: 5′-ATA-GAA-GAA-GGG-AAA-TAG-CAA-ATG-G-3′<br>Reverse: 5′-TTA-ACG-ATG-GAG-TCC-AAG-TTC-TGG-3′	95°C (30 sec), 56°C (30 sec), and<br>72°C (1 min) for 30 cycles; 232 bp
GAPDH	Forward: 5′-GAA-GGT-GAA-GGT-CGG-AGT-3′<br>Reverse: 5′-GAA-GAT-GGT-GAT-GGG-ATT-TC-3′	95°C (30 sec), 56°C (40 sec), and<br>72°C (40 sec) for 25 cycles; 220 bp
